# Relationship between coronary hyper-intensive plaques identified by cardiovascular magnetic resonance and clinical severity of acute coronary syndrome

**DOI:** 10.1186/s12968-021-00706-7

**Published:** 2021-02-25

**Authors:** Wen Liu, Sijing Wu, Zhenjia Wang, Yanni Du, Zhaoyang Fan, Li Dong, Yonghe Guo, Yi Liu, Xiaoming Bi, Jing An, Yujie Zhou, Wei Liu, Debiao Li, Wei Yu, Yibin Xie

**Affiliations:** 1 Department of Radiology, Anzhen Hospital, Affiliated to Capital Medical University, 2 Anzhen Road, ChaoYang District, Beijing, 100029 China; 2 Department of Radiology, Peking University Cancer Hospital & Institute, 52 Fucheng Road, Hai Dian District, Beijing, 100142 China; 3 Department of Cardiology, Beijing AnZhen Hospital, Affiliated to Capital Medical University, Anzhen Road, ChaoYang District, Beijing, 100029 China; 4 Cedars-Sinai Medical Center, Biomedical Imaging Research Institute, Los Angeles, CA USA; 5 Department of Biomedical Sciences, Cedars-Sinai Medical Center, Biomedical Imaging Research Institute, Los Angeles, CA USA; 6 MR R&D, Siemens Healthineers, Los Angeles, CA USA; 7 MR Collaborations NE Asia, Siemens Healthineers, Beijing, China

**Keywords:** Cardiovascular magnetic resonance, Coronary hyper-intense plaque (CHIP), Acute coronary syndrome, Optical coherence tomography, Thrombus

## Abstract

**Background:**

Coronary hyper-intense plaque (CHIP) detected on T1-weighted cardiovascular magnetic resonance (CMR) has been shown to associate with vulnerable plaque features and worse outcomes in low- and intermediate-risk populations. However, the prevalence of CHIP and its clinical significance in the higher-risk acute coronary syndrome (ACS) population have not been systematically studied. This study aims to assess the relationship between CHIP and ACS clinical severity using intracoronary optical coherence tomography (OCT) as the reference.

**Methods:**

A total of 62 patients with known or suspected coronary artery disease were prospectively enrolled including a clinically diagnosed ACS group (n = 50) and a control group with stable angina pectoris (n = 12). The ACS group consisted of consecutive patients including unstable angina pectoris (n = 27), non-ST-segment-elevation myocardial infarction (non-STEMI) (n = 8), and ST-segment-elevation myocardial infarction (STEMI) (n = 15), respectively. All patients underwent non-contrast coronary CMR to determine the plaque-to-myocardium signal intensity ratio (PMR).

**Results:**

Among the four groups of patients, a progressive increase in the prevalence of CHIPs (stable angina, 8%; unstable angina, 26%; non-STEMI, 38%; STEMI, 67%; p = 0.009), and PMR values (stable angina, 1.1; unstable angina, 1.2; non-STEMI, 1.3; STEMI, 1.6; median values, P = 0.004) were observed. Thrombus (7/8, 88% vs. 4/22, 18%, p = 0.001) and plaque rupture (5/8, 63% vs. 2/22, 9%, p = 0.007) were significantly more prevalent in CHIPs than in plaques without hyper-intensity. Elevated PMR was associated with high-risk plaque features including plaque rupture, thrombus, and intimal vasculature. A positive correlation was observed between PMR and the number of high-risk plaque features identified by OCT (r = 0.44, p = 0.015).

**Conclusions:**

The prevalence of CHIPs and PMR are positively associated with the disease severity and high-risk plaque morphology in ACS.

## Background

Acute coronary syndrome (ACS) accounts for > 50% of sudden cardiac death cases and typically arises from an indolent atherosclerosis process [1]. Abrupt rupture of plaque fibrous cap with subsequent thrombosis is considered the most common mechanism of ACS [2]. Early detection of high-risk coronary lesions prone to rupture may lead to improved risk-stratification and potential prevention of ACS. In the past decade, T1-weighted (T1w) cardiovascular magnetic resonance (CMR) was introduced for the morphological assessment of high-risk coronary plaques. Several clinical studies found that coronary hyper-intense plaque (CHIP) on T1w CMR indicates thrombus and possibly intra-plaque hemorrhage (IPH). For instance, Jansen et al. showed that CHIPs on T1w imaging could correctly identify intraluminal thrombus of coronary arteries in 9 of 10 patients suffering from ACS, within 24 to 72 h after symptom onset [4]. Matsumoto et al. demonstrated that intraluminal CHIPs on T1w imaging were independently associated with thrombus and the presence of microvessels, while intra-wall CHIPs were associated with macrophages and probable IPH, based on the reference of optical coherence tomography (OCT) [3].

Despite the capacity to detect coronary plaques with high-risk characteristics, conventional CMR protocols have drawbacks such as long acquisition time, limited anatomical coverage, and lack of anatomical reference, which prevented its wide clinical application. Recently, a more advanced CMR acquisition strategy, coronary atherosclerosis T1w characterization with integrated anatomical reference (CATCH), was introduced using motion-corrected interleaved data acquisition to provide dark-blood T1w images along with bright-blood anatomical reference images simultaneously [5]. The preliminary clinical study showed that CHIPs detected on pre- and post-contrast CATCH are associated with the various high-risk plaque features on OCT [5]. A recent ex vivo validation study with histopathological reference showed high sensitivity and specificity for identifying IPH using CATCH [6]. Nonetheless, previous investigations primarily focused on low- and intermediate-risk populations. The prevalence of CHIP and its clinical relevance in the higher-risk ACS population have yet to be systematically evaluated. The aim of this study is to assess the potential association between plaque hyper-intensity on CATCH and the clinical severity of ACS using intracoronary OCT as the reference for high-risk plaques.

## Methods

The study was approved by the institutional ethics review board, and all study subjects provided informed consent prior to participation.

### Study population

**Fig. 1:**
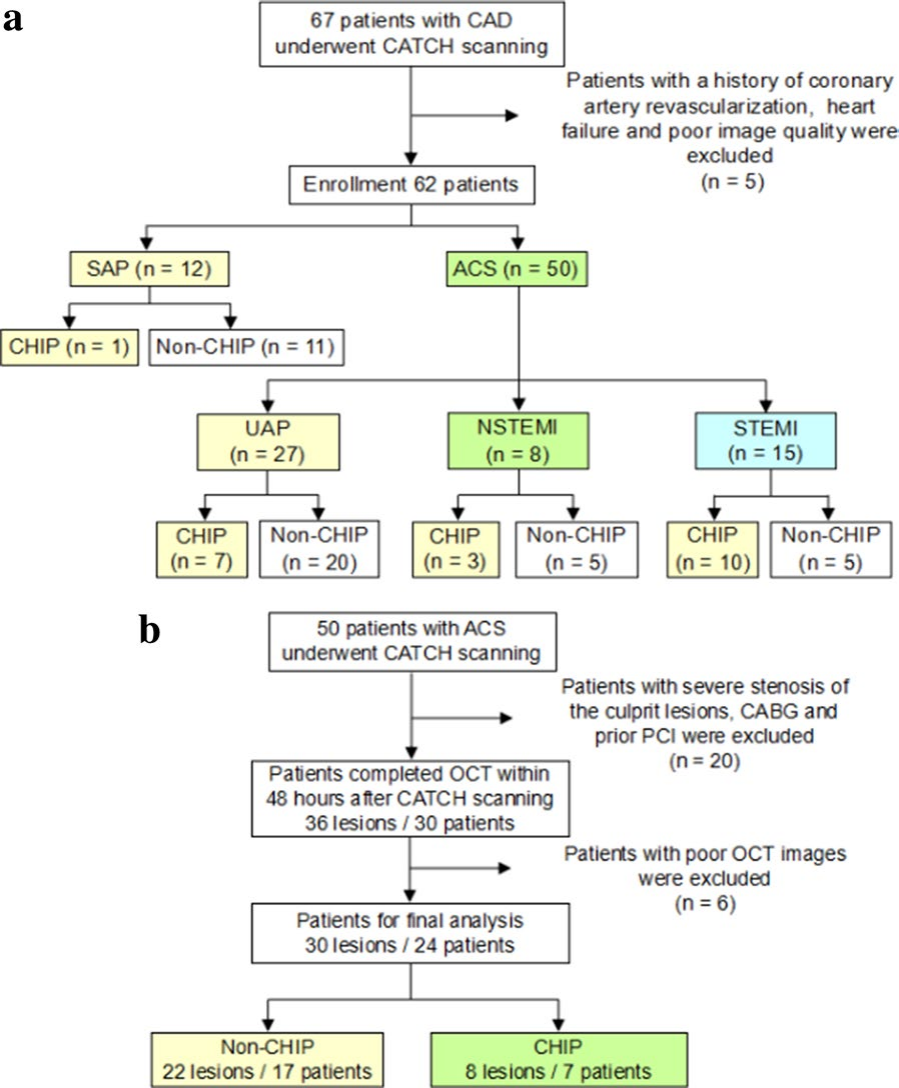
Flowchart of the study design for patient recruitment and inclusion. **a** A total of 67 patients with coronary artery disease (CAD) in the Department of Cardiology were prospectively enrolled. Overall, 50 patients with acute coronary syndrome (ACS) and 12 patients with stable angina were included in the final analysis. Proven ACS was classified into 3 subgroups, including unstable angina (n = 27), non-ST elevation myocardial infarction (NSTEMI) (n = 8), and ST elevation myocardial infarction (STEMI) (n = 15). CHIPs were observed in 1 (8%) stable angina, 7 (26%) unstable angina, and 3 (38%) NSTEMI, and 10 (67%) STEMI patients, respectively. **b** Of the 50 ACS patients, 24 patients (including 30 lesions; 8 lesions with CHIPs and 22 lesions with non-CHIPs) were included in the final analysis

Between November 2016 and April 2018, 67 patients (59 men; 57 ± 11 years) with known or suspected coronary artery disease (CAD) were prospectively enrolled from the Department of Cardiology. The study cohort consisted of 55 consecutive patients with clinically diagnosed ACS, and 12 additionally recruited patients with stable angina pectoris for control. Patients with a history of coronary artery revascularization (percutaneous coronary intervention, coronary artery bypass graft surgery, and/or thrombolytic therapy, n = 2), heart failure (n = 1) and poor image quality (n = 2) were subsequently excluded from the study, after which a total of 62 patients were included in the image analysis. The ACS group consisted of 3 clinically defined subgroups: (1) unstable angina pectoris (n = 27), defined as ischemia with proven angina pectoris at rest, without elevated high-sensitivity cardiac troponin I (hs-cTnI) levels or elevated ST on initial electrocardiogram (ECG); (2) non-ST-segment-elevation myocardial infarction (non-STEMI) (n = 8), defined as ischemia with proven angina pectoris and elevated hs-cTnI levels, while no evidence of ST-segment elevation on initial ECG [7,8]; (3) ST-segment-elevation myocardial infarction (STEMI) (n = 15), defined as ischemia with proven angina pectoris and elevated hs-cTnI levels, in addition to ST elevation or new left bundle-branch block [9]. Patients with STEMI and non-STEMI were studied 7–20 days after symptom onset as patients within 7 days of symptom onset receivedtherapy in the emergency room and therefore were enrolled. The control group (n = 12) included patients with stable angina, defined as ischemia with unchanged exertional angina pectoris for > 2 months. All patients enrolled underwent non-contrast CATCH examinations. (Fig. 1a).

The majority of patients with ACS (n = 30) underwent pre-interventional OCT based on clinical needs within 48 h after CMR. Patients unsuitable for OCT examination included ones with severe stenosis of the culprit lesions (n = 8), planned coronary artery bypass grafting (n = 5), and prior percutaneous coronary intervention (n = 7). Additionally, patients (n = 6) who had poor OCT image quality were excluded. Eventually, a total of 24 patients (30 lesions) with ACS were included in the comparison between CATCH and OCT for plaque features (Fig. 1b).

### CATCH imaging and analysis

CATCH imaging was performed on a clinical 3 T CMR Scanner (MAGNETOM Verio, Siemens Healthineers, Erlangen, Germany) with a 12-channel body matrix coil. The procedures used for CMR imaging acquisition were described previously ^[[[[5]]]]^. Briefly, three-dimensional whole heart pre-contrast CATCH images were acquired with fat-suppressed, inversion recovery prepared spoiled gradient sequence. T1w dark-blood images and bright-blood anatomical reference images were acquired in the same scan in an interleaved fashion which allowed for joint motion compensation. The main imaging parameters of CATCH included: field of view (FOV), 322 × 242 × 108 mm^3^; acquired spatial resolution, 1.3 × 1.3 × 1.3 mm^3^ (interpolated to 0.7 × 0.7 × 1.3 mm^3^); TE/TR, 2.38/4.8 ms; flip angle, 15°; inversion time, 430 to 580 ms, inversion pulse applied every other heartbeat; number of averages, 1; bandwidth, 443 Hz/pixel; acquisition time, approximately 12 min depending on heart rate. A patient-specific inversion time was determined in each case using a look-up table, which was precalculated based on different heart rates and acquisition window durations by Bloch equation simulations.

CATCH images were reviewed by two experienced radiologists blinded to OCT findings and patient information. As previously described [3,5,11], T1w dark-blood plaque images were fused with anatomical reference images on a clinical image post-processing workstation (Leonardo, Siemens Healthineers) by the Fusion functionality. Dark-blood T1w images, bright-blood anatomical reference images, and Fusion images were displayed simultaneously to localize coronary arteries and plaques.

Each target lesion location was determined carefully by comparing coronary angiography images with the CATCH bright-blood images, using anatomical landmarks such as the distance to side branches and degree of coronary artery stenosis as the reference. Quantification of CHIP was based on plaque-to-myocardium signal intensity ratio (PMR), defined as the maximal signal intensity of the plaque region divided by the mean signal intensity of adjacent ventricular myocardium. PMR was determined immediately after a measurement process similar to that described by previous studies using T1w images [5,11]. To ensure the inclusion of the entire lesion, region of interest was defined by manually tracing the outer boundary of each vascular segment of interest before extending to the contiguous imaging slices. Plaques with PMR > 1.4 were defined as CHIPs, and those with PMR ≤ 1.4 considered non-CHIPs, according to the criteria determined by its prognosis [12]. For patients with multi-vessel lesions, one culprit lesion was used in each patient for patient-based analysis, which was determined by the ECG test results, findings on invasive coronary artery angiography and other relevant clinical information. For patients with 2 or more lesions on the same coronary artery in terms of selecting the lesion of interest for analysis, the culprit lesion, which was the most serious on invasive coronary angiography, was used for analysis. The analysis of T1w images took approximately 1 h for each case.

To evaluate the potential impact of disease severity on the image quality, a 4-point image quality score was used for the assessment of CATCH coronary image quality in the four different CAD groups using a widely adopted method described in a previous coronary CMR study [10]. Briefly, 0 denotes that the coronary artery is invisible and 1 to 4 indicate increasing degrees of vessel delineation with 4 as the best. A mean image quality score was calculated for all enrolled subjects of the four different CAD groups. As the dark-blood images heavily suppressed the signal from lumen and normal vessel wall, only the bright-blood images were used in the evaluation to represent the quality of the entire dataset which was acquired in an interleaved fashion. One-way analysis of variance (ANOVA) was used for comparing the four different CAD groups for the image quality, and post hoc tests were used for comparing the image quality of each two groups.

### OCT acquisition and analysis

A total of 24 patients (30 lesions) underwent clinically indicated pre-interventional OCT imaging, using a frequency-domain system as previously described [13–15].

OCT images were analyzed by 2 experienced cardiologists (WL, and SW, with 10 and 6 years of experience, respectively) who were blinded to CMR data. The following characteristics of high-risk plaques on OCT images were assessed qualitatively along a culprit lesion segment of 10-mm-long (5 mm proximal and 5 mm distal to the culprit lesion site with the smallest luminal cross-sectional area) [3], using previously described criteria for OCT plaque features [13–15]: (1) calcification, defined as a signal-poor region with well-delineated sharp borders; (2) plaque rupture, defined as an interruption of the internal elastic lamina followed by cavity formation in the plaque; (3) thrombus, defined as a mass protruding into the lumen with irregularly low or high backscattering signal variance. And thrombus was further classified into two subtypes: white and red thrombus, depending on the backscattering signal intensity. The intraluminal protrusion with low backscattering signal was defined as white thrombus, otherwise red thrombus; (4) microvessels, defined as a trajectory or black hole within the internal elastic lamina of the vessel; (5) thin-cap fibroatheroma (TCFA), defined as a lipid-rich region with fibrous cap thickness ≤ 65 mm; (6) macrophage infiltration, defined as punctate signal-rich spots resulting in high backscattering signal variance; (7) lipid-rich plaques, defined as signal-poor region with diffuse borders in ≥ 2 quadrants in any of the plaque images. If the initial diagnosis between 2 observers did not match, a second reading was performed to reach consensus.

### Statistical analysis

Continuous, normally distributed parameters were reported as mean ± SD, and non-normally distributed ones as median and interquartile range. Categorical data were reported as count and percentage. A comparison of variables between the two groups was performed by unpaired t-test or Kruskal–Wallis test for non-normally distributed data. The Chi-square test or Fisher exact test was applied for categorical variables as appropriate. For multiple categorical variables, if there were statistically significant differences in the results, further comparisons were made between groups. One-way analysis of variance (ANOVA) was used for multiple group comparisons, such as PMR comparison and the image quality comparison in different types of patients. Post hoc analysis was then used for a comparison between groups, which was a supplement of the ANOVA analysis. Cohen’s Kappa value was used to quantify intra- and inter-observer agreement detected CHIP, and intraclass correlation coefficients with 95% confidence intervals (CI) for agreement measured PMR. The relationship between the number of vulnerable plaques detected on OCT and PMR values was evaluated by Pearson correlation analysis. All statistical analyses were conducted in SPSS (version 19.0, Statistical Package for the Social Sciences, International Business Machines, Inc., Armonk, New York, USA). P < 0.05 was considered statistically significant.

## Results

### Patient-based analysis

**Fig. 2:**
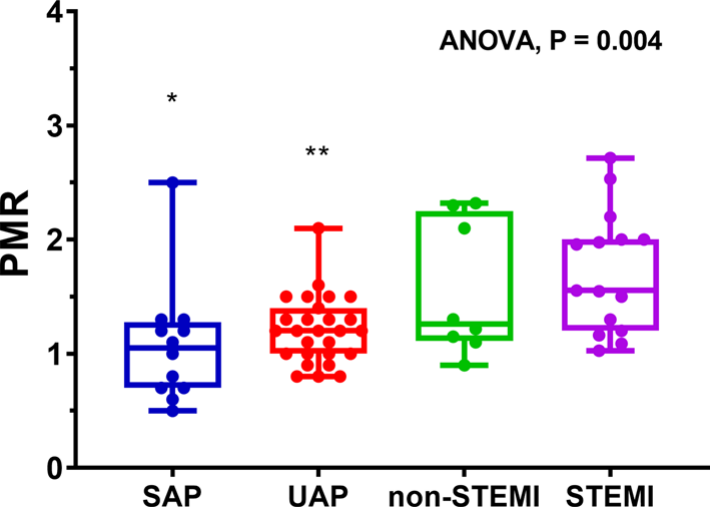
A comparison of plaque hyper-intensity (in plaque to myocardium signal intensity ratio (PMR)) between different severities of coronary artery disease. Results are expressed as a box and whisker diagram with minimum, first quartile, median, third quartile, maximum and all individual data points. A progressive increase in PMR was observed among patients with stable angina, unstable angina, non-STEMI, and STEMI (p = 0.004, ANOVA). Post hoc tests showed PMR values of STEMI group was significantly higher than stable angina group (P = 0.007) (*) and unstable angina groups (P = 0.006) (**). However, it has not reached statistic differences between other groups

The clinical characteristics and angiographic findings of the 62 patients who were included in the analyses are summarized in Table 1. There was no significant difference in their basic clinical characteristics or the localization of culprit vessels between the four groups. The ANOVA result shows the image quality of CMR coronary was similar among all four groups (stable angina, 2.92 ± 0.67; unstable angina, 2.89 ± 0.64; non-STEMI, 2.88 ± 0.35; STEMI, 3.00 ± 0.85; P = 0.96). Post hoc tests show the image quality between each two groups was also not significantly different (P = 1.0). High intra- and inter-observer agreement was observed for the detection of CHIP (k = 0.859 and 0.762, respectively, P < 0.001), and the assessment of PMR (intraclass correlation coefficient = 0.96 (95% CI 0.93–0.98) and 0.95 (95% CI 0.91–0.97), respectively, P < 0.001). Among all patients, 21 (34%) had CHIPs with a PMR cutoff value of 1.4. The prevalence of CHIP lesions in the four groups of patients was different, there was a progressive increase in the frequency of CHIP lesions among four groups (stable angina, 8%; unstable angina, 26%; non-STEMI, 38%; STEMI, 67%; P = 0.009). Further comparisons between groups showed the frequency of CHIP lesions in patients with STEMI was higher than stable angina (P = 0.005) and UAP (P = 0.02), however, it has not reached statistic differences between patients with other groups (STEMI and non-STEMI, P = 0.221; stable angina and unstable angina, P = 0.394; stable angina and non-STEMI, P = 0.255; unstable angina and non-STEMI, P = 0.661). Among patients with stable angina, unstable angina, non-STEMI, and STEMI, the median, 25th percentile, and 75th percentile PMR values were progressively higher (stable angina, 1.1, 0.7, and 1.3; unstable angina, 1.2, 1.0, and 1.4; non-STEMI, 1.3, 1.1, and 2.3; STEMI, 1.6, 1.2, and 2.0; P = 0.004) (Fig. 2). Post hoc tests showed PMR values of STEMI group was significantly higher than stable angina group (P = 0.007) and unstable angina group (P = 0.006). However, it has not reached statistic differences between other groups (STEMI and non-STEMI, P = 0.821; stable angina and unstable angina, P = 1.0; stable angina and non-STEMI, P = 0.92; unstable angina and non-STEMI, P = 1.0).

**Table 1:** Subject clinical characteristics and invasive angiographic findings

	Stable angina (n = 12)	ACS (n = 50)			P value
		Unstable angina (n = 27)	Non-STEMI (n = 8)	STEMI (n = 15)	
PMR	1.1 (0.7–1.3)	1.2 (1.0–1.4)	1.3 (1.1–2.3)	1.6 (1.2–2.0)	0.004
CHIPs	1 (8%)	7 (26%)	3 (38%)	10 (67%)	0.009
Age, yrs	53 ± 14	56 ± 12	60 ± 9	50 ± 9	0.177
Male	11 (92%)	23 (85%)	7 (88%)	14 (93%)	0.856
Diabetes mellitus	3 (25%)	11 (41%)	2 (25%)	4 (27%)	0.663
Smoking	3 (25%)	9 (33%)	3 (38%)	9 (60%)	0.246
Dyslipidemia	2 (17%)	3 (11%)	0 (0%)	2 (13%)	0.699
Systolic blood pressure, mm Hg	125 ± 9	127 ± 16	128 ± 13	119 ± 12	0.294
Diastolic blood pressure, mm Hg	75 ± 9	73 ± 10	71 ± 9	77 ± 10	0.498
Culprit Vessel					0.524
Left anterior descending	6 (50%)	18 (67%)	4 (50%)	5 (33%)	
Left circumflex	2 (17%)	2 (7%)	1 (13%)	5 (33%)	
Right coronary artery	4 (33%)	6 (22%)	3 (37%)	4 (27%)	
Left main coronary artery	0 (0%)	1 (4%)	0 (0%)	1 (7%)	

Values are mean ± SD, median (interquartile range), or n (%). *ACS* acute coronary syndrome, *PMR* plaque-to-cardiac muscle signal intensity ratio, *CHIP* coronary hyper-intensive plaque

### Vessel-based analysis

The relationships between CHIP detection by CATCH and plaque morphology detected by OCT and angiographic findings in patients who completed invasive coronary angiography and pre-interventional OCT are summarized in Table 2. Of the 30 lesions which were examined by OCT, 8 (27%) were classified as CHIPs and 22 (73%) were non-CHIPs. The frequency of thrombus (P = 0.001) and plaque rupture (P = 0.007) was significantly higher in CHIPs than non-CHIPs. Thrombus was detected in 7 (88%) and 4 (18%) CHIP and non-CHIP lesions, respectively. Of the 7 CHIPs with confirmed thrombus on OCT, 5 and 2 were red and white thrombi, respectively. Of the 4 non-CHIPs, 2 and 2 lesions had red and white thrombi, respectively. The diameter stenosis in CHIP lesions was higher than that of non-CHIP lesions (P = 0.020). The differences between the two lesion types in the frequency of TCFA, lipid-rich plaques, macrophage accumulation, calcification or intimal vasculature did not reach statistical significance.

**Table 2:** Comparison of high-risk plaque features detected by invasive angiography and intracoronary optical coherence tomography (OCT) in CHIPs and non-CHIPs

	CHIPs (n = 8)	Non-CHIPs (n = 22)	P value
Lipid length, mm	9.4 (8.3–10.0)	8.9 (5.5–10.0)	0.400
Max lipid arc, degree	302.9 (281.8–332.6)	276.7 (224.3–313.5)	0.185
Lipid-rich plaque	4 (50%)	10 (45%)	1.000
TCFA	0 (0%)	4 (18%)	0.550
Plaque rupture	5 (63%)	2 (9%)	0.007
Calcification	2 (25%)	11 (50%)	0.407
Thrombus	7 (88%)	4 (18%)	0.001
Red thrombus	5 (71%)	2 (50%)	
White thrombus	2 (29%)	2 (50%)	
Macrophage accumulation	2 (25%)	10 (45%)	0.419
Intimal vasculature	3 (38%)	4 (18%)	0.345
Percent diameter stenosis	72.7 ± 19.7	56.7 ± 14.2	0.020

Values are n (%) or median (interquartile range). *CHIP* coronary hyper-intensive plaque, *OCT* optical coherence tomography, *TCFA* thin-cap fibroatheroma

**Fig. 3:**
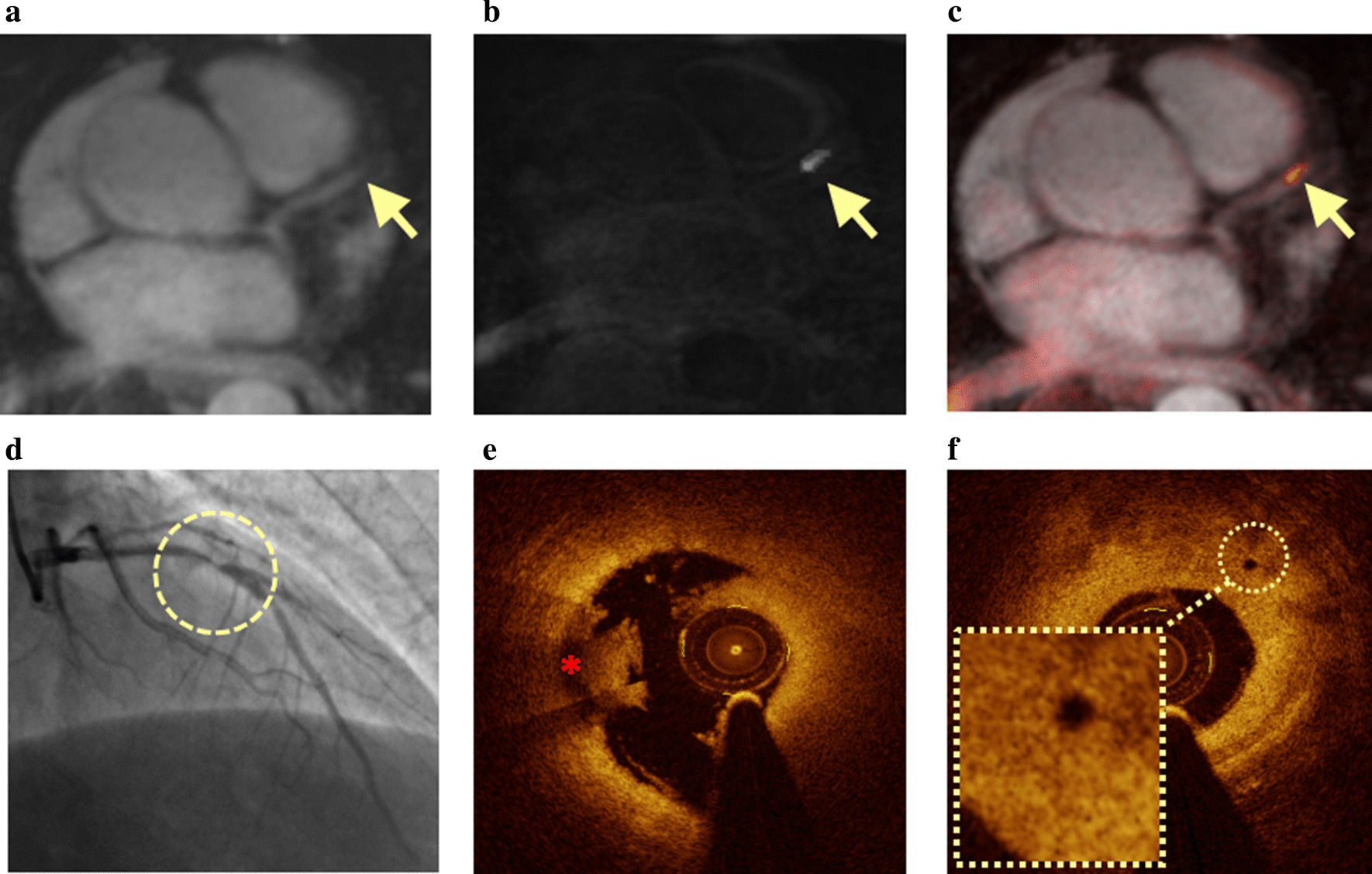
A representative ACS case with a CHIP on CATCH compared with invasive coronary imaging. A 58-year-old male subject with unstable angina pectoris. Coronary plaque images acquired with CATCH are shown (**a**, bright blood image; **b**, dark blood image; **c**, fusion image). CATCH dark blood image (**b**) and fusion image (**c**) showed high signal intensity in the proximal left anterior descending coronary artery with a PMR of 1.5 (arrows). Invasive coronary angiography (**d**) showed high-grade stenosis in the segment corresponding to the hyper-intensive plaque on the CATCH images (circle). OCT examination (**e** and **f**) showed intraluminal thrombus (red star) and microvessel in the corresponding lesion (circle)

A representative patient with ACS and a CHIP lesion on CATCH is shown in Fig. 3. Coronary angiography showed significant stenosis in the proximal left anterior descending coronary artery. CATCH dark blood T1w and fusion images showed hyper-intensity with PMR = 1.5, in the area corresponding to the severe stenosis. Intracoronary OCT examination showed the presence of intraluminal thrombus and microvessel at the corresponding vessel segment.

**Fig. 4:**
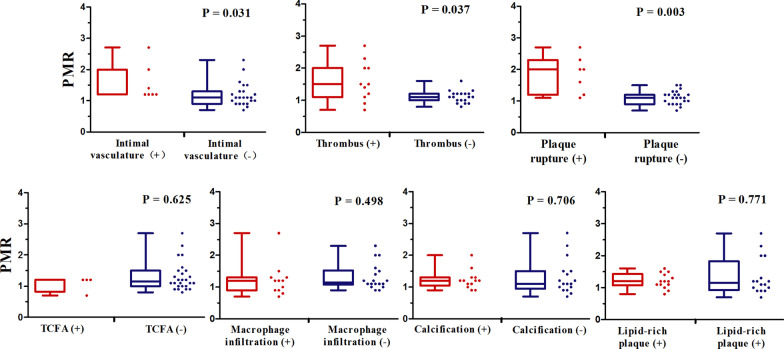
Relationship between high-risk plaque features and plaque to myocardium ratio (PMR) in ACS. High-risk plaque features such as plaque rupture (P = 0.003), thrombus (P = 0.037) and intimal vasculature (P = 0.031) were associated with significantly higher PMR; no significant differences were found in other plaque features including the presence of TCFA (P = 0.625), lipid-rich plaques (P = 0.771), absence of calcification (P = 0.706) and macrophage infiltration (P = 0.498)

The relationship between plaque morphology detected by OCT and PMR obtained by CATCH is shown in Fig. 4. High-risk plaque features, such as plaque rupture (P = 0.003), thrombus (P = 0.037) and intimal vasculature (P = 0.031) were associated with significantly higher PMR. No significant difference in PMR was found in other plaque types, including the presence of TCFA (P = 0.625), lipid-rich plaques (P = 0.771), absence of calcification (P = 0.706) and macrophage infiltration (P = 0.498).

Pearson correlation analysis revealed a moderate but significant positive correlation between PMR and the number of high-risk plaque features observed on OCT (P = 0.015, r = 0.44).

## Discussion

In this study, we demonstrated the feasibility of integrating CATCH, a novel CMR technique, into the clinical workflow of managing patients with ACS. To the best of our knowledge, the results in this study are the first evidence illustrating the differential CMR plaque morphologies in the three subtypes of ACS. Our data supported a clear association between plaque hyper-intensity on CATCH and the clinical severity of ACS as confirmed by the high-risk plaque features detected by OCT.

Previous studies showed that CHIPs detected by T1w CMR were associated with intraluminal thrombus in patients with stable and unstable angina [16]. The findings in our study illustrated that the majority of CHIPs in the ACS population were directly associated with different types of thrombi. We observed thrombi in 88% of the lesions classified as CHIPs in ACS, which was higher than the 75% rate previously reported in a study on patients with stable and unstable angina [3]. We also observed plaque rupture in 63% of CHIPs, which was also higher than the range of 19 to 42% in previous studies of intermediate-risk groups [3]. On the other hand, 4 patients from the current study had thrombus detected by OCT but no CHIP on CATCH. After ruling out the possibility of poor image quality in these cases, we postulate that such mismatch is a possible result of the variation of the age and volume of thrombus under in vivo conditions, manifesting as different signal intensities on T1W images [17]. The time delay between CATCH and invasive exams may also allow the thrombus to relocate or change in size. Moreover, we showed that the frequency of intimal vasculature (CHIPs, 38%; non-CHIPs, 18%) and the absence of calcification (CHIPs, 75%; non-CHIPs, 50%) were higher in CHIPs than non-CHIPs, although with no statistically significance, likely due to the small sample size of the study.

Recent studies suggested that PMR can serve as a quantitative indicator of high-risk plaques. Xie et al. [5] showed a positive correlation between PMR from both non-contrast and contrast-enhanced CATCH and the number of high-risk plaque features on OCT in patients with stable angina. Matsumoto et al. [18] also demonstrated that elevated PMR was associated with an accumulation of high-risk plaque morphology. The results in our present study were partially aligned with the previous findings as we observed a positive association between elevated PMR with plaque rupture, thrombus, and intimal vasculature observed on OCT. However, no significant relationship was found between PMR and other plaque features, including the presence of TCFA, lipid-rich plaque, absence of calcification and macrophage infiltration. Our data suggest that the coronary lesions in the ACS population, particularly in the myocardial infarction groups, may involve a different pathological status compared with the lower risk stable or unstable angina pectoris populations as previously studied. We hypothesize that the more prominent findings on thrombus than TCFA in our study is a result of a higher frequency of abrupt rupture of plaque fibrous cap with subsequent thrombosis, a well-known mechanism for ACS [2].

### Clinical implications

Patients with ACS typically have higher incidence rates of vulnerable plaque features such as intimal vasculature, TCFA, plaque rupture, and intracoronary thrombus, compared with patients with stable angina, according to previous invasive imaging studies [19]. Intracoronary thrombus is commonly detected in STEMI by OCT [20]. Our current study showed that the majority of CHIPs in the ACS population were directly associated with thrombus and plaque rupture according to the reference of OCT. Moreover, PMR values of STEMI group was significantly higher than the stable angina group and unstable groups. In addition, we also showed a stepwise increase existed in PMR values within the three subgroups of ACS with increasing severity, although with no statistically significance and requiring further validation via a much larger sample size. These findings demonstrated that CATCH, a noninvasive imaging technique can provide lesion-specific risk assessment regarding the disease status in the ACS population.

The results from this study support the hypothesis that non-contrast CATCH may potentially aid the diagnosis and improved the risk-stratification of ACS. The presence of CHIP with higher PMR value provides imaging-based evidence regarding the presence and location of high-risk coronary plaques. Since ACS is an unstable disease with a high fatality rate, early detection of high-risk coronary lesions prior to intervention would inform lesion-specific treatment strategy. For patients who show borderline clinical presentations for ACS and did not receive immediate reperfusion therapy, additional diagnostic information provided by CATCH, which is fast and noninvasive, might further risk-stratify patients who would be otherwise only passively monitored. With the advantage of whole-heart coverage, fine isotropic resolution and simultaneously acquired dark-blood and bright-blood images, upon further validation, CATCH would supplement conventional CMR protocols to provide a “one-stop” evaluation of both myocardium and coronary arteries in CAD patients with different disease severity.

### Limitations and future directions

First, the sample size of the study was relatively small due to the logistical challenges associated with this specific study cohort. Nonetheless, the results obtained in the study were sufficient to support the main findings, even though the statistical power was inadequate for evaluating the entire spectrum of vulnerable coronary plaque features. Second, the results in this study may suffer from selection bias as a result of the limitation in recruitment. Patients with infarction who were within 7 days after symptom onset were treated immediately in the emergency room, therefore were not able to enroll in this study. On the other hand, patients with infarction who enrolled in the study underwent CMR 7 to 20 days after the initial onset of symptoms. The characteristics of coronary plaques could have evolved during the interval of acute disease progression, and a possible mismatch may occur between observed and representative plaque morphology in the respective populations. Third, in this study, signal intensities of culprit lesions detected by CATCH were compared with the morphological features of plaques as identified by OCT without further verification by pathology. Even though OCT is considered as the standard in invasive plaque characterization, its accuracy and reproducibility are not fully established for all plaque features. For example, macrophage infiltration by OCT is not quantified or rigorously validated. Although the direct relationship between CHIPs and coronary intra-plaque hemorrhage cannot be validated due to the lack of in vivo reference, previous histopathologic studies of carotid atherosclerotic plaques strongly supported the direct link between intraplaque hemorrhage and high-intensity signal on T1w images [6,21–23]. We used PMR of 1.4 as the threshold for determining CHIPs, which was adopted based on the findings by Noguchi et al. [11]. The dichotomization is based on achieving the optimal prognostic value, not by specific plaque features. The best threshold of PMR for differentiating each type of plaque feature may vary therefore requires further investigation in a larger cohort. Future analysis of CHIPs may include the integration of hyperintensive volume to further improve the prognostic value of T1w imaging [24].

## Conclusions

This study demonstrated that CHIPs detected by non-contrast CATCH in patients with ACS are associated with the clinical severity of the disease. Quantitative plaque hyperintensity by PMR is progressively more elevated in more severe ACS subtypes, as well as in lesions with an increasing number of high-risk plaque features on OCT. Upon further validation, CATCH may serve as a noninvasive imaging method for aiding lesion-specific diagnosis and guiding treatment strategies in the ACS populations.

## Data Availability

The datasets used and analyzed in the current study are available from the corresponding authors upon reasonable request.

## Abbreviations

ACS: Acute coronary syndrome
CAD: Coronary artery disease
CATCH: Coronary atherosclerosis T1-weighted characterization with integrated anatomical reference
CHIP: Coronary high intensity plaque
CI: Confidence intervals
CMR: Cardiovascular magnetic resonance
ECG: Electrocardiogram
FOV: Field of view
hs-cTnI: High-sensitivity cardiac troponin I
IPH: Intra-plaque hemorrhage
NSTEMI: Non-ST elevation myocardial infarction
OCT: Optical coherence tomography
PMR: Plaque-to-myocardium signal intensity ratio
STEMI: ST-segment-elevation myocardial infarction
T1W: T1-weighted
TCFA: Thin-cap fibroatheroma
